# Editorial: Organization of the White Matter Anatomy in the Human Brain

**DOI:** 10.3389/fnana.2019.00085

**Published:** 2019-09-18

**Authors:** Silvio Sarubbo, Laurent Petit

**Affiliations:** ^1^Division of Neurosurgery, Structural and Functional Connectivity Lab Project, Azienda Provinciale per i Servizi Sanitari, Trento, Italy; ^2^Groupe d'Imagerie Neurofonctionnelle, Institut des Maladies Neurodégératives (IMN)-UMR5293-CNRS, CEA, Université de Bordeaux, Bordeaux, France

**Keywords:** white matter, structural anatomy, functional anatomy, brain processing, neurosurgery, tractography, functional MRI, direct electrical stimulation

Between nineteenth and twentieth centuries, neurosciences experienced the first sharing of experiences and competences between the world of brain anatomy and clinics. The improvements in the knowledge of human white matter (WM) anatomy provided the natural background to the structural definition of a wide spectrum of clinical syndromes. This “disconnection” experience was the first field of strict integration between the WM anatomical and clinical skills, and constituted the hard core for the development of the modern neurosciences over the last century (Catani and ffytche, 2005).

While the second half of twentieth century has seen the neurophysiology taking a front role in the definition of the physiological and physio-pathological processing of brain circuitries, the last decade has definitively brought neuroimaging into the world of neuroscience. The functional magnetic resonance imaging (fMRI) and diffusion-weighted MRI (DWI) tractography have successively opened a new era for a better understanding of functional and structural anatomy of the human brain (Le Bihan and Johansen-Berg, 2012; Smith et al., 2013).

In particular, DWI-based tractography was the first tool allowing the exploration of human WM *in vivo* with an unprecedented level of details, and it shed a new light in the knowledge of the brain anatomy that became, finally, more accessible (Jeurissen et al., 2019). Beyond the technical aspects related to the continuous necessary improvement of this approach (Maier-Hein et al., 2017), tractography produced a conceptual revolution leading that the wiring diagram of brain connections regained a center scene of neuroscience research. Such a revolution was not only in research but also in the clinical and neurosurgical domains and opened the “connectome” era (Sporns, 2013).

The fields of neuroanatomy, neuroimaging, neurophysiology and clinical researches are currently closer as never before. In fact, two decades of exploration of brain structure and functional processing with an unprecedented level of sensitivity opened new challenges. Among others, the research for a ground truth in structural anatomy is definitely the most impressive, especially considering the basic and conceptual consequences of that in assessing a reliable knowledge of brain processing, clinics and plasticity. This is what the vast majority of the articles in this Research Topic highlight by describing association WM pathways (Bao et al.; David et al.; Panesar et al.), cortico-striatal Cacciola et al. and cortico-thalamic (Maffei et al.; Roddy et al.; Sun et al.) projection pathways.

Many techniques are now available for the exploration of structural anatomy of human WM. At macrostructural level, besides the millimetric and widespread power of resolution of DWI, polarized light imaging (PLI, see in this Research Topic the report of Schmitz et al.) and polarization sensitive optical coherence tomography (PSOCT) are emerging techniques with an unprecedented level of accuracy in defining at micrometer resolution the structural anatomy of WM in humans (Caspers and Axer, 2017). Even, microdissection of fixated specimens (i.e., modified Klingler's preparation, Sarubbo et al., 2015b) experienced a renewed interest in confirming previous evidences or re-defining course and terminations of associative, commissural and projection pathways in human and non-human primate brain (Sarubbo et al., 2013, 2016, 2019; Fernandez-Miranda et al., 2015; De Benedictis et al., 2016; Hau et al., 2016, 2017; Wang et al., 2016; Maffei et al., 2018). Limitations and strength points of each of these techniques allowed to partially reduce the technical gap for clarifying and distinguishing among the open questions in the field of human WM neuroanatomy. Nevertheless, no single technique can be considered alone as fully reliable for a whole and wide range study of brain connections.

In this scenario, the multimodal integration of evidences provided by different techniques is the most reliable approach for the investigation of the human connectome. Confirming the reliability of the different approaches and integrating structural and functional multimodal evidences, from both *in-* and *ex-vivo* studies, provide more reliable data as well as some technical and conceptual refinements of each approach. This virtuous loop already allowed:

- to propose the existence of a new association bundle in the human brain (David et al.);
- to integrate for the first time direct electrical WM stimulation evidences with DWI tractography (Sarubbo et al., 2015a) for exploring the functional organization of the complex human speech articulatory network (Zaca et al., 2018);
- to obtain tractography reconstructions anatomically-driven by the evidences provided by microdissection (De Benedictis et al., 2016; Hau et al., 2016, 2017);
- the first extractions of quantitative data from microdissection techniques, or the integration of these pure anatomical data in a common radiological space with an unprecedented level of definition due to photogrammetric 3D-models and post-mortem MRI acquisition (De Benedictis et al., 2018).

Each of the 9 articles presented in this Research Topic is fully in line with fascinating current debates on the definition, organization, terminology, and conceptualization of the anatomy of human WM.

## Author Contributions

All authors listed have made a substantial, direct and intellectual contribution to the work, and approved it for publication.

### Conflict of Interest Statement

The authors declare that the research was conducted in the absence of any commercial or financial relationships that could be construed as a potential conflict of interest.

## References

[B1] Caspers S. Axer M. (2017). Decoding the microstructural correlate of diffusion MRI. NMR Biomed. 32:e3779. 10.1002/nbm.3779 28858413

[B2] Catani M. ffytche D. H. (2005). The rises and falls of disconnection syndromes. Brain 128, 2224–2239. 10.1093/brain/awh622 16141282

[B3] De Benedictis A. Nocerino E. Menna F. Remondino F. Barbareschi M. Rozzanigo U.. (2018). Photogrammetry of the human brain: a novel method for three-dimensional quantitative exploration of the structural connectivity in neurosurgery and neurosciences. World Neurosurg. 115, e279–e291. 10.1016/j.wneu.2018.04.036 29660551

[B4] De Benedictis A. Petit L. Descoteaux M. Marras C. E. Barbareschi M. Corsini F. (2016). New insights in the homotopic and heterotopic connectivity of the frontal part of the human corpus callosum revealed by microdissection and diffusion tractography. Hum. Brain Mapp. 37, 4718–4735. 10.1002/hbm.23339 27500966 PMC6867471

[B5] Fernandez-Miranda J. C. Wang Y. Pathak S. Stefaneau L. Verstynen T. Yeh F. C. (2015). Asymmetry, connectivity, and segmentation of the arcuate fascicle in the human brain. Brain Struct. Funct. 220, 1665–1680. 10.1007/s00429-014-0751-7 24633827

[B6] Hau J. Sarubbo S. Houde J. C. Corsini F. Girard G. Deledalle C.. (2017). Revisiting the human uncinate fasciculus, its subcomponents and asymmetries with stem-based tractography and microdissection validation. Brain Struct. Funct. 222, 1645–1662. 10.1007/s00429-016-1298-6 27581617

[B7] Hau J. Sarubbo S. Perchey G. Crivello F. Zago L. Mellet E. (2016). Cortical terminations of the inferior fronto-occipital and uncinate fasciculi: stem-based anatomical virtual dissection. *Front*. Neuroanat. 10:58 10.3389/fnana.2016.00058 PMC4877506 27252628

[B8] Jeurissen B. Descoteaux M. Mori S. Leemans A. (2019). Diffusion MRI fiber tractography of the brain. NMR Biomed. 32:e3785. 10.1002/nbm.3785 28945294

[B9] Le Bihan D. Johansen-Berg H. (2012). Diffusion MRI at 25: Exploring brain tissue structure and function. Neuroimage 61, 324–341. 10.1016/j.neuroimage.2011.11.006 22120012 PMC3683822

[B10] Maffei C. Jovicich J. De Benedictis A. Corsini F. Barbareschi M. Chioffi F.. (2018). Topography of the human acoustic radiation as revealed by *ex vivo* fibers micro-dissection and *in vivo* diffusion-based tractography. Brain Struct. Funct. 223, 449–459. 10.1007/s00429-017-1471-6 28866840

[B11] Maier-Hein K. H. Neher P. F. Houde J. C. Côté M. A. Garyfallidis E. Zhong J.. (2017). The challenge of mapping the human connectome based on diffusion tractography. Nat. Commun. 8:1349. 10.1038/s41467-017-01285-x 29116093 PMC5677006

[B12] Sarubbo S. De Benedictis A. Maldonado I. L. Basso G. Duffau H. (2013). Frontal terminations for the inferior fronto-occipital fascicle: anatomical dissection, DTI study and functional considerations on a multi-component bundle. Brain Struct. Funct. 218, 21–37. 10.1007/s00429-011-0372-3 22200882

[B13] Sarubbo S. De Benedictis A. Merler S. Mandonnet E. Balbi S. Granieri E.. (2015a). Towards a functional atlas of human white matter. Hum. Brain Mapp. 36, 3117–3136. 10.1002/hbm.22832 25959791 PMC6869563

[B14] Sarubbo S. De Benedictis A. Merler S. Mandonnet E. Barbareschi M. Dallabona M.. (2016). Structural and functional integration between dorsal and ventral language streams as revealed by blunt dissection and direct electrical stimulation. Hum. Brain Mapp. 37, 3858–3872. 10.1002/hbm.23281 27258125 PMC6867442

[B15] Sarubbo S. De Benedictis A. Milani P. Paradiso B. Barbareschi M. Rozzanigo U.. (2015b). The course and the anatomo-functional relationships of the optic radiation: a combined study with ‘post mortem' dissections and ‘*in vivo'* direct electrical mapping. J. Anat. 226, 47–59. 10.1111/joa.12‘54 25402811 PMC4313898

[B16] Sarubbo S. Petit L. De Benedictis A. Chioffi F. Ptito M. Dyrby T. B. (2019). Uncovering the inferior fronto-occipital fascicle and its topological organization in non-human primates: the missing connection for language evolution. Brain Struct. Funct. 224, 1553–1567. 10.1007/s00429-019-01856-2 30847641

[B17] Smith S. M. Vidaurre D. Beckmann C. F. Glasser M. F. Jenkinson M. Miller K. L.. (2013). Functional connectomics from resting-state fMRI. Trends Cogn. Sci. 17, 666–682. 10.1016/j.tics.2013.09.016 24238796 PMC4004765

[B18] Sporns O. (2013). The human connectome: Origins and challenges. Neuroimage 80, 53–61. 10.1016/j.neuroimage.2013.03.023 23528922

[B19] Wang X. Pathak S. Stefaneanu L. Yeh F. C. Li S. Fernandez-Miranda J. C. (2016). Subcomponents and connectivity of the superior longitudinal fasciculus in the human brain. Brain Struct. Funct. 221, 2075–2092. 10.1007/s00429-015-1028-5 25782434

[B20] Zaca D. Corsini F. Rozzanigo U. Dallabona M. Avesani P. Annicchiarico L. (2018). Whole-brain network connectivity underlyin+g the human speech articulation as emerged integrating direct electric stimulation, resting state fMRI and tractography. Front. Hum. Neurosci. 12:405 10.3389/fnhum.2018.00405 30364298 PMC6193478

